# Separate and combined effects of famine exposure and menarche age on metabolic syndrome among the elderly: a cross-sectional study in China

**DOI:** 10.1186/s12905-023-02737-x

**Published:** 2023-11-14

**Authors:** Congzhi Wang, Jiazhi Wang, Rui Wan, Ting Yuan, Liu Yang, Dongmei Zhang, Xiaoping Li, Haiyang Liu, Lin Zhang

**Affiliations:** 1 Department of Internal Medicine Nursing, School of Nursing, Wannan Medical College, An Hui Province, Wuhu City, 241000 P.R China; 2 Sports Institute, Chi Zhou College, Education Park, Chi Zhou City, An Hui Province People’s Republic of China; 3 Business School, Yunnan University of Finance and Economics, 237 Longquan Road, Kunming City, Yun Nan Province, People’s Republic of China; 4 Obstetrics and Gynecology Nursing, School of Nursing, Wannan Medical College, 22 Wenchang West Road, Higher Education Park, Wuhu City, An Hui Province People’s Republic of China; 5 Department of Pediatric Nursing, School of Nursing, Wannan Medical College, 22 Wenchang West Road, Higher Education Park, Wuhu City, An Hui Province People’s Republic of China; 6 Department of Emergency and Critical Care Nursing, School of Nursing, Wannan Medical College, 22 Wenchang West Road, Higher Education Park, Wuhu City, An Hui Province People’s Republic of China; 7 Student Health Center, Wannan Medical College, 22 Wenchang West Road, Higher Education ParkAn Hui Province, Wuhu City, People’s Republic of China

**Keywords:** Famine exposure, Menarche age, Metabolic syndrome, The elderly, Cross-sectional study, China, Regression analysis

## Abstract

**Background:**

Epidemiological studies have revealed multiple risk factors for metabolic syndrome. However, there are no consistent findings on the association between famine exposure, age at menarche, and the prevalence of metabolic syndrome. This cross-sectional study aimed to reveal the individual and combined effects of famine exposure and age at menarche on the prevalence of metabolic syndrome among elderly women.

**Methods:**

Four thousand seven hundred seventy participants between 60 and 93 years of age were selected from the China Health and Retirement Longitudinal Study. Statistical differences between the baseline characteristics of famine exposure, age at menarche, and metabolic syndrome were evaluated using the t-test, F-test, and Chi-square test. Three multivariable-adjusted logistic regression models were used to test the association between famine exposure, age of menarche, and the odds ratio of metabolic syndrome.

**Results:**

Two thousand one hundred ninety-eight (46.08%) participants had metabolic syndrome, while 2572 (53.92%) participants did not. Furthermore, 3068 (64.32%) women reported onset of menarche under 15 years of age, while 1702 (35.68%) women reported onset of menarche above 16 years of age. Regarding the separate association of famine exposure and age of menarche with metabolic syndrome, in model three, the adolescence/adulthood famine exposure group vs. no famine exposure group odds ratio was 2.45 (95% CI 2.02, 2.97), and the older than 16 years vs. younger than 15 years group odds ratio was 1.23 (95% CI 1.09, 1.39), which was the highest odds ratio among the three models. Regarding the combined association of famine exposure and age of menarche with metabolic syndrome, in model three, among the age of menarche ≤ 15 years group, the adolescence/adulthood famine exposure vs. no famine exposure group odds ratio was 2.45 (95% CI: 1.91, 3.14); among the menarche age ≥ 16 years group, the adolescence/adulthood famine exposure stages vs. exposed group odds ratio was 3.27 (95% CI: 2.44, 4.38), which was the highest odds ratio among the three models.

**Conclusion:**

These findings suggested that famine exposure and age at menarche, either separately or in combination, were positively associated with the prevalence of metabolic syndrome among older women.

## Introduction

Metabolic Syndrome (MetS) is defined as a cluster of conditions, including visceral adiposity, low high-density lipoprotein (HDL) cholesterol levels, high fasting triglyceride levels, high blood pressure, and elevated fasting plasma glucose concentration [1–3]. MetS also increases the risk of other illnesses such as cardiovascular and cerebrovascular diseases, diabetes, and cancer [4–7]. The reported prevalence of MetS was 35% in the USA and 31% in Spain [8], with a rapid increase from 21.3% to 33.9% between 2009 and 2019 in China [9], affecting millions of people and attracting public attention for nearly a decade. The main causes of MetS are obesity, puberty, insulin resistance, and hormone secretion disturbances [1, 10–12]. Age at menarche age is one of the predisposing factors for metabolic syndrome. Menarche is characterized by signs of female maturity and marks the onset of monthly hormone secretion and reproductive life. Previous studies have shown that menarche initiation results in early puberty and metabolic dysregulation [13–17]. Several studies have demonstrated the association between age at menarche and the prevalence of MetS; however, the results remain controversial. Accumulating evidence suggests that menarche age is inversely associated with MetS prevalence [18, 19], although some studies identified a U-shaped association between menarche and MetS [20]. Conversely, a study conducted in Bangladesh, showed that delayed menarche reduced the prevalence of metabolic syndrome [21]. Other studies found no association between the prevalence of MetS and age at menarche [22, 23].

The thrifty genotype hypothesis postulates that individuals experiencing famine are more inclined to conserve energy than to consume it, even if malnutrition caused by hunger has been eliminated [24, 25]. According to this hypothesis, early-life malnutrition was beneficial for survivors in the short term, with adverse effects persisting until adulthood, mainly affecting the body's metabolic rate [25]. Both cross-sectional and cohort studies have revealed a separate influence of famine exposure on the risk of MetS; however, the conclusions are inconsistent. Most studies have found that famine exposure during early life is strongly associated with an increased prevalence of MetS, particularly fetal exposure, and exposure during infancy [26–29]. Studies on famine exposure in adolescence and adulthood are limited. Conversely, studies in the Netherlands and China have shown no association between famine exposure and MetS [9, 30].

Few studies have investigated the association between age at menarche, famine exposure at different stages of development, and the prevalence of MetS. To clarify and expand the etiology of MetS, we examined the individual and combined effects of famine exposure and age at menarche on the prevalence of MetS among elderly females. Additionally, further expansion of the MetS risk factors and evidence of preventive measures against MetS are needed.

## Patients and methods

### Baseline patient characteristics

**Fig. 1:**
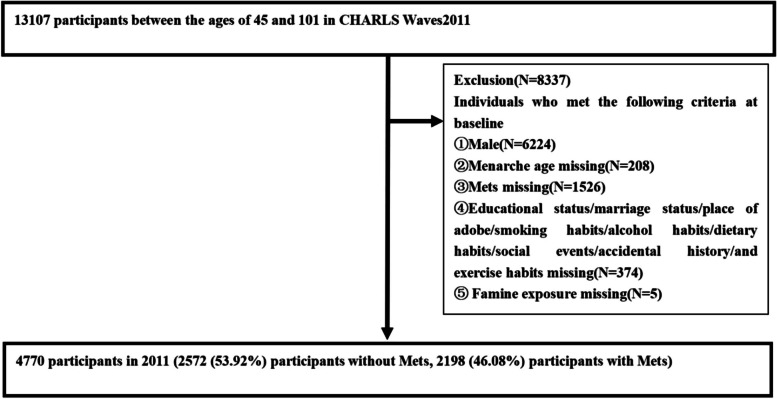
The flowchart of the participants enrolled in the study

Four thousand seven hundred seventy samples were selected from the China Health and Retirement Longitudinal Study (CHARLS). Participant age in the CHARLS was 58.64 ± 9.23 [mean ± standard deviation; range: 45 to 93 years]. The age of menarche of all the participants was 16.29 ± 2.19 [mean ± standard deviation age = year]. We included participants who matched the criteria at baseline: the participants were born between 1921 and 1966. Out of 13,107 participants, we excluded 6,224 males, 1,526 missing MetS data, 5 missing famine exposure data, 208 missing ages at menarche data, and 374 missing data on educational status, marriage status, place of adobe, smoking habits, alcohol habits, dietary habits, social events, accidental history, and exercise habits. Thus, we analyzed data from a total of 4,470 participants, including 2,572 (53.92%) diagnosed without MetS, and 2,198 (46.08%) diagnosed with MetS. Figure 1 represents the flowchart of the study participants, follow-up, and loss to follow-up. The following variables used in this paper are derived from our previous research [31–35], and include: educational status, measured as illiteracy, less than grade school, junior school, and above vocational institute; marriage status, categorized as spinsterhood and married; place of adobe, categorized as rural and city; smoking habits, categorized as no, being used to smoking, and current smoking; alcohol habits were categorized as no, less than once per month, and more than once per month; dietary habits were classified as less than 2 times per day, 3 times per day, and more than 4 times per day; social events was categorized into no or yes; accidental history was divided into no or yes; exercise habits were categorized into no exercise, less than regular exercise, and regular exercise; famine exposure was categorized into no exposure, fetal exposure, childhood exposure, and adolescence/adulthood exposure; menarche age was categorized as ≤ 15 years and ≥ 16 years; MetS was categorized into no and yes.

### Sample calculation

This cross-sectional study was conducted by random sampling of the target population. An adequate sample size is necessary to precisely determine the association between diseases and related factors. The prevalence of MetS was 30.85% in China, the index of α was 0.05, and d was calculated as 0.02 × p. The following calculation yielded 897 samples, and our study met the minimum sample size requirement for this study [44].

$$n=\frac{{Z}_{1-a/2}^{2}\times p\left(1-p\right)}{{d}^{2}}$$

### Measurements

MetS was defined according to the following criteria based on the interim statement of the International Diabetes Federation, which is widely used in previous studies [9, 36, 37]: (1) abdominal obesity, defined as a waist circumference (WC) of more than 80 cm for Asian females; (2) low level of HDL cholesterol, defined as a value less than 50 mg/dL; (3) high fasting triglyceride levels, defined as a value greater than 150 mg/dL; (4) hypertension, defined as systolic blood pressure over 130 mmHg and diastolic blood pressure over 85 mmHg; (5) high fasting plasma glucose concentration, defined as a concentration greater than 100 mg/dL.

### Age at menarche

The median age at menarche was 16 years, categorized as ≤ 15 or ≥ 16 years.

### Exposure age and exposure according to stages of development

According to the birth year of each participant, the team consulted previously relevant published papers [31–35, 38–42]. The participants were divided into four groups: the no exposure group, including female participants born between 1963 and 1966; the fetal exposure group, including female participants born between 1959 and 1962; the childhood exposure group, including female participants born between 1949 and 1958; and the adolescence/adulthood exposure group, including female participants born between 1921 and 1948 [28].

### Statistical analysis

Statistical analysis was performed using SPSS software (version 22.0; IBM Corp., Armonk, NY, USA). The data are presented as numbers and percentages (categorical data) and were used to evaluate the classification variables (educational status, marital status, place of adobe, smoking habits, alcohol habits, dietary habits, social events, accidental history, exercise habits, famine exposure, age at menarche, and MetS). Among these groups, differences according to famine exposure (no exposure, fetal exposure, childhood exposure, and adolescent/adult exposure), menarche age (≤ 15 years and ≥ 16 years), and MetS (with or without MetS) were assessed using the chi-square test or t-test (categorical data). Adjusted odds ratios (ORs) were used to calculate the separate and combined effects of MetS prevalence using a multivariate-adjusted logistic regression model. The model was constructed by logistic regression analysis using the Forward: LR method (Forward Stepwise Regression based on Maximum Likelihood Estimation). Three models were used to calculate the prevalence of MetS among different subgroups. Model one was adjusted for age, model two was adjusted for educational status, marital status, and place of abode; model three was adjusted for educational status, marital status, place of abode, smoking habits, alcohol habits, dietary habits, social events, accidental history, and exercise habits.

## Results

Table 1 shows the baseline characteristics of the participants, including 4470 participants, comprising 786 (16.48%) participants with no exposure to famine, 611 (12.81%) with fetal exposure, 1904 (39.92%) with childhood exposure, and 1469 (30.39%) with adolescent/adulthood exposure. Among the four groups, significant differences existed in these basic characteristics, including educational status, marital status, smoking habits, alcohol habits, dietary habits, social events, exercise habits, age at menarche, and MetS (*P* < 0.05). Furthermore, no significant differences were found in the place of abode (*P* = 0.740) and accidental history (*P* = 0.089).

**Table 1:** Characteristics of participants in the study by level of famine exposure (*N* = 4770)

**Variables**	No exposure	Fetal exposure	Childhood exposure	Adolescence/ adult exposure	Total	χ2	*P*
**N**	786	611	1904	1469	4770		
**Educational Status**						580.421	0.000
Illiteracy	133(16.92)	164(26.84)	855(44.91)	850(57.86)	2002(41.97)		
less than grade school	570(72.52)	336(54.99)	942(49.47)	582(39.62)	2430(50.94)		
Junior school	56(7.12)	100(16.37)	83(4.36)	10(0.68)	249(5.22)		
Above vocational institute	27(3.44)	11(1.8)	24(1.26)	27(1.84)	89(1.87)		
**Marriage Status**						510.708	0.000
Spinsterhood	23(2.93)	32(5.24)	175(9.19)	462(31.45)	692(14.51)		
Married	763(97.07)	579(94.76)	1729(90.81)	1007(68.55)	4078(85.49)		
**Place of abode**						1.252	0.740
Rural	493(62.72)	394(64.48)	1206(63.34)	951(64.74)	3044(63.82)		
City	293(37.28)	217(35.52)	698(36.66)	518(35.26)	1726(36.18)		
**Smoking habits**						64.336	0.000
No	758(96.44)	577(94.44)	1765(92.7)	1296(88.22)	4396(92.16)		
Being used to smoking	3(0.38)	6(0.98)	31(1.63)	54(3.68)	94(1.97)		
Current smoking	25(3.18)	28(4.58)	108(5.67)	119(8.1)	280(5.87)		
**Alcohol habits**						20.779	0.002
No	690(87.79)	526(86.09)	1676(88.03)	1295(88.16)	4187(87.78)		
Less than once per month	47(5.98)	43(7.04)	102(5.36)	49(3.34)	241(5.05)		
More than once per month	49(6.23)	42(6.87)	126(6.62)	125(8.51)	342(7.17)		
**Dietary habits**						13.839	0.031
less than 2 times per day	120(15.27)	78(12.77)	217(11.4)	212(14.43)	627(13.14)		
3 times per day	661(84.1)	525(85.92)	1660(87.18)	1234(84)	4080(85.53)		
more than 4 times per day	5(0.64)	8(1.31)	27(1.42)	23(1.57)	63(1.32)		
**Social events**						27.62	0.000
No	357(45.42)	262(42.88)	953(50.05)	792(53.91)	2364(49.56)		
Yes	429(54.58)	349(57.12)	951(49.95)	677(46.09)	2406(50.44)		
**Accidental history**						6.52	0.089
No	748(95.17)	567(92.8)	1762(92.54)	1362(92.72)	4439(93.06)		
Yes	38(4.83)	44(7.2)	142(7.46)	107(7.28)	331(6.94)		
**Exercises habits**						15.572	0.016
No exercise	468(59.54)	349(57.12)	1141(59.93)	954(64.94)	2912(61.05)		
Less than regular exercise	158(20.1)	126(20.62)	378(19.85)	261(17.77)	923(19.35)		
Regular exercise	160(20.36)	136(22.26)	385(20.22)	254(17.29)	935(19.6)		
**Menarche age**						87.521	0.000
≤ 15 years	425(54.07)	348(56.96)	1239(65.07)	1056(71.89)	3068(64.32)		
≥ 16 years	361(45.93)	263(43.04)	665(34.93)	413(28.11)	1702(35.68)		
**Metabolic syndrome**						74.101	0.000
No	512(65.14)	348(56.96)	1028(53.99)	684(46.56)	2572(53.92)		
Yes	274(34.86)	263(43.04)	876(46.01)	785(53.44)	2198(46.08)		

Table 2 shows the differences between the two groups according to age at menarche (≤ 15 years and ≥ 16 years). Overall, 3068 (64.32%) participants reported an age at menarche of less than 15 years and 1702 (35.68%) reported an age at menarche of more than 16 years. Significant differences were found in educational status, place of abode, smoking habits, social events, famine exposure, and MetS (*P* < 0.05). Non-significant differences were observed for marital status (*P* = 0.060), alcohol consumption habits (*P* = 0.173), dietary habits (*P* = 0.102), accidental history (*P* = 0.229), and exercise habits (*P* = 0.827).

**Table 2:** Characteristics of participants in the study categorized by menarche age (*N* = 4770)

**Variables**	≤ 15 years	≥ 16 years	Total	χ2	*P*
**N**	3068	1702	4770		
**Educational Status**				107.805	0.000
Illiteracy	1438(46.87)	564(33.14)	2002(41.97)		
less than grade school	1470(47.91)	960(56.4)	2430(50.94)		
Junior school	121(3.94)	128(7.52)	249(5.22)		
Above vocational institute	39(1.27)	50(2.94)	89(1.87)		
**Marriage Status**				3.537	0.060
Spinsterhood	467(15.22)	225(13.22)	692(14.51)		
Married	2601(84.78)	1477(86.78)	4078(85.49)		
**Place of abode**				28.676	0.000
Rural	2043(66.59)	1001(58.81)	3044(63.82)		
City	1025(33.41)	701(41.19)	1726(36.18)		
**Smoking habits**				11.58	0.003
No	2804(91.4)	1592(93.54)	4396(92.16)		
Being used to smoking	75(2.44)	19(1.12)	94(1.97)		
Current smoking	189(6.16)	91(5.35)	280(5.87)		
**Alcohol habits**				3.504	0.173
No	2673(87.13)	1514(88.95)	4187(87.78)		
Less than once per month	165(5.38)	76(4.47)	241(5.05)		
More than once per month	230(7.5)	112(6.58)	342(7.17)		
**Dietary habits**				4.573	0.102
less than 2 times per day	385(12.55)	242(14.22)	627(13.14)		
3 times per day	2637(85.95)	1443(84.78)	4080(85.53)		
more than 4 times per day	46(1.5)	17(1)	63(1.32)		
**Social events**				11.259	0.001
No	1576(51.37)	788(46.3)	2364(49.56)		
Yes	1492(48.63)	914(53.7)	2406(50.44)		
**Accidental history**				1.445	0.229
No	2845(92.73)	1594(93.65)	4439(93.06)		
Yes	223(7.27)	108(6.35)	331(6.94)		
**Exercises habits**				0.379	0.827
No exercise	1873(61.05)	1039(61.05)	2912(61.05)		
Less than regular exercise	600(19.56)	323(18.98)	923(19.35)		
Regular exercise	595(19.39)	340(19.98)	935(19.6)		
**Famine exposure**				87.521	0.000
No exposure	425(13.85)	361(21.21)	786(16.48)		
Fetal exposure	348(11.34)	263(15.45)	611(12.81)		
Childhood exposure	1239(40.38)	665(39.07)	1904(39.92)		
Adolescence/adult exposure	1056(34.42)	413(24.27)	1469(30.8)		
**Metabolic syndrome**				13.557	0.000
No	1715(55.9)	857(50.35)	2572(53.92)		
Yes	1353(44.1)	845(49.65)	2198(46.08)		

Table 3 shows the differences between participants without MetS (*n* = 2572, 53.92%) and those with MetS (*n* = 2198, 46.08%). Significant differences were found between the two groups in place of abode, smoking habits, alcohol habits, exercise habits, famine exposure, and age at menarche (*P* < 0.05). In addition, there were no significant differences in any characteristics, including educational status (*P* = 0.128), marital status (*P* = 0.796), dietary habits (*P* = 0.054), and accidental history (*P* = 0.276).

**Table 3:** Characteristics of participants in the study categorized by metabolic syndrome status (*N* = 4770)

**Variables**	Without metabolic syndrome	Metabolic syndrome	Total	χ2	*P*
**N**	2572	2198	4770		
**Educational status**				5.692	0.128
Illiteracy	1084(42.15)	918(41.77)	2002(41.97)		
less than grade school	1301(50.58)	1129(51.36)	2430(50.94)		
Junior school	147(5.72)	102(4.64)	249(5.22)		
Above vocational institute	40(1.56)	49(2.23)	89(1.87)		
**Marriage status**				0.067	0.796
Spinsterhood	370(14.39)	322(14.65)	692(14.51)		
Married	2202(85.61)	1876(85.35)	4078(85.49)		
**Place of abode**				40.797	0.000
Rural	1747(67.92)	1297(59.01)	3044(63.82)		
City	825(32.08)	901(40.99)	1726(36.18)		
**Smoking habits**				7.036	0.030
No	2383(92.65)	2013(91.58)	4396(92.16)		
Being used to smoking	38(1.48)	56(2.55)	94(1.97)		
Current smoking	151(5.87)	129(5.87)	280(5.87)		
**Alcohol habits**				12.435	0.002
No	2219(86.28)	1968(89.54)	4187(87.78)		
Less than once per month	141(5.48)	100(4.55)	241(5.05)		
More than once per month	212(8.24)	130(5.91)	342(7.17)		
**Dietary habits**				5.849	0.054
less than 2 times per day	359(13.96)	268(12.19)	627(13.14)		
3 times per day	2173(84.49)	1907(86.76)	4080(85.53)		
more than 4 times per day	40(1.56)	23(1.05)	63(1.32)		
**Social events**				11.481	0.001
No	1333(51.83)	1031(46.91)	2364(49.56)		
Yes	1239(48.17)	1167(53.09)	2406(50.44)		
**Accidental history**				1.185	0.276
No	2384(92.69)	2055(93.49)	4439(93.06)		
Yes	188(7.31)	143(6.51)	331(6.94)		
**Exercises habits**				8.448	0.015
No exercise	1579(61.39)	1333(60.65)	2912(61.05)		
Less than regular exercise regular exercise	524(20.37) 469(18.23)	399(18.15) 466(21.2)	923(19.35) 935(19.6)		
**Famine exposure**				74.101	0.000
No exposure	512(19.91)	274(12.47)	786(16.48)		
Fetal exposure	348(13.53)	263(11.97)	611(12.81)		
Childhood exposure	1028(39.97)	876(39.85)	1904(39.92)		
Adolescence/adult exposure	684(26.59)	785(35.71)	1469(30.8)		
**Menarche age**				13.557	0.000
≤ 15 years	1715(66.68)	1353(61.56)	3068(64.32)		
≥ 16 years	857(33.32)	845(38.44)	1702(35.68)		

Table 4 shows the separate associations between famine exposure, menarche age, and the prevalence of MetS in females. Compared with the no exposure to famine group, MetS prevalence was higher in all subgroups. Furthermore, in multivariable model one, the most significant increase in the odds ratio was observed for the adolescence/adulthood exposure stage (OR 2.14; 95% CI 1.79, 2.56) (*P* < 0.05); in multivariable-adjusted model two, after adjustment for educational status, marriage status, and place of abode, the highest odds of prevalence in MetS were observed for the adolescence/adulthood exposure stage (OR 2.38; 95% CI 1.96, 2.88) (*P* < 0.05). Additionally, in multivariable-adjusted model three, after adjusting for educational status, marital status, place of abode, smoking habits, alcohol habits, dietary habits, social events, accidental history, and exercise habits, the highest odds ratio of prevalence in MetS was observed in the adolescence/adulthood exposure stage (OR 2.45; 95% CI 2.02, 2.97) (*P* < 0.05). Furthermore, compared with the group with menarche age below 15 years, the odds ratios of prevalence in MetS were observed for menarche age above 16 years among the three models (Model one: OR = 1.25, 95% CI 1.11,1.41; model two: OR = 1.24, 95% CI 1.10,1.40; model three: OR = 1.23, 95% CI 1.09,1.39).

**Table 4:** Separate associations of famine exposure, menarche age with the prevalence of metabolic syndrome (*N* = 4770)

Famine exposure	Model one^a^	Model two^b^	Model three^c^
No exposure	1.00(reference)	1.00(reference)	1.00(reference)
Fetal exposure	1.41(1.14,1.75)	1.44(1.16,1.79)	1.44(1.15,1.79)
Childhood exposure	1.59(1.34,1.89)	1.65(1.38,1.97)	1.67(1.40,1.99)
Adolescence/adult exposure	2.14(1.79,2.56)	2.38(1.96,2.88)	2.45(2.02,2.97)
***P* for trend**	0.000	0.000	0.000
**Menarche age**			
≤ 15 years	1.00(reference)	1.00(reference)	1.00(reference)
≥ 16 years	1.25(1.11,1.41)	1.24(1.10,1.40)	1.23(1.09,1.39)

^a^Unadjusted; age-adjusted by design;

^b^Adjusted for educational status, marriage status, and place of adobe

^c^Adjusted for educational status, marriage status, place of adobe, smoking habits, alcohol habits, dietary habits, social events, accidental history, and exercise habits

Table 5 shows the combined association of famine exposure and age at menarche with MetS prevalence in females. Compared to the combination of non-exposed famine stage and age at menarche under 15 years, the prevalence of MetS tended to be higher in all subgroups, and in the multivariable model, the most significant increase in odds ratio was observed for the adolescence/adulthood famine exposure stage and menarche age above 16 years (OR 2.97; 95% CI 2.24, 3.94) (*P* < 0.05). In multivariable-adjusted model two, after adjusting for educational status, marital status, and place of abode, the highest odds ratio of MetS was observed for the adolescence/adulthood exposure stage and menarche age above 16 years (OR 3.18; 95% CI 2.37, 4.25) (*P* < 0.05). In addition, after adjusting for educational status, marital status, place of residence, smoking habits, alcohol habits, dietary habits, social events, accidental history, and exercise habits, the highest odds ratio of prevalence in MetS was observed for the adolescence/adulthood exposure stage and menarche age above 16 years in model three (OR 3.27; 95% CI 2.44, 4.38) (*P* < 0.05).

**Table 5:** Combined associations of famine exposure and menarche age with the prevalence of metabolic syndrome (*N* = 4770)

**Famine exposure**	**Metabolic syndrome odds ratio (95%** *CI***)**					
	**Model one**^**a**^		**Model two**^**b**^		**Model three**^**c**^	
	Menarche age		Menarche age		Menarche age	
Female	≤ 15 years	≥ 16 years	≤ 15 years	≥ 16 years	≤ 15 years	≥ 16 years
No exposure	1.00(reference)	1.26(0.94,1.69)	1.00(reference)	1.19(0.89,1.61)	1.00(reference)	1.21(0.9,1.63)
Fetal exposure	1.45(1.08,1.95)	1.75(1.27,2.39)	1.47(1.09,1.97)	1.69(1.23,2.33)	1.48(1.10,1.99)	1.69(1.23,2.33)
Childhood exposure	1.55(1.23,1.96)	2.26(1.75,2.91)	1.58(1.25,2.00)	2.22(1.72,2.87)	1.61(1.27,2.04)	2.24(1.73,2.89)
Adolescence/adult exposure	2.19(1.73,2.78)	2.97(2.24,3.94)	2.35(1.84,3.01)	3.18(2.37,4.25)	2.45(1.91,3.14)	3.27(2.44,4.38)
*P* for trend	0.000	0.000	0.000	0.000	0.000	0.000

^a^Unadjusted; age-adjusted by design

^b^Adjusted for educational status, marriage status, and place of adobe

^c^Adjusted for educational status, marriage status, place of adobe, smoking habits, alcohol habits, dietary habits, social events, accidental history, and exercise habits

## Discussion

This cross-sectional study explored the separate and combined effects of famine exposure and age at menarche on the prevalence of MetS in females. This study found that the females exposed to famine antenatally and during childhood and adolescence/adulthood had a higher risk of MetS than those in the no-exposure group. After adjusting for confounding factors, an association between the subgroups (childhood and adolescence/adulthood exposure) was still observed. Additionally, compared with the group of participants aged ≤ 15 years at menarche, those aged ≥ 16 years had a higher prevalence of MetS. Furthermore, compared to the participants with age at menarche ≤ 15 years and with no famine exposure, those with a later age at menarche (≥ 16 years) and famine exposure (particularly adolescence/adulthood exposure) had the highest prevalence of MetS. After adjusting for confounding factors, the association between the different groups remained. Overall, the study showed that famine exposure and age at menarche had a combined positive association with a higher prevalence of MetS than individual factors.

For the fetal and childhood exposure stages, our study found that females with famine exposure had a higher prevalence of MetS, which is hypothesized to cause damage to tissues and organs that continue into adulthood and lead to various chronic diseases [24, 43]. Numerous studies conducted in China have shown that famine exposure in early life is correlated with an increased risk of MetS, which is consistent with our study [44, 45]. Research on the Ethiopian Famine also demonstrated a positive association between famine exposure and increased risk of MetS [46]. A meta-analysis showed that people exposed to famine in fetal life and infancy had a higher risk of developing MetS than those not exposed to famine [8]. It has also been demonstrated that early malnutrition persistently alters cholesterol synthesis and plasma cholesterol concentrations in animal models, eventually leading to hypercholesterolemia and metabolic disorders [47]. However, this association was not observed by Sun and de Rooij SR in their studies on adolescence and prenatal famine exposure, respectively [9, 30], which was inconsistent with the findings of our study. These discrepancies may be the result of differences in the statistical methods, basic demographic characteristics, and regional characteristics. Several potential mechanisms explain this positive association. Famine exposure is associated with damage to the lipid profile [48], methylation of the imprinted insulin-like growth factor 2 (IGF2) gene [49], DNA methylation [50], visceral adipose dysfunction, and beta cell dysfunction [28], all of which contribute to the occurrence of MetS, as mentioned above. Furthermore, hunger experienced at an early age can lead to behavioural changes, such as more smoking, less physical activity, and a preference for eating fatty foods, which are the result of a mismatch between famine malnutrition, and abundant food options later in life [51–55]. The thrifty phenotype hypothesis postulates that early exposure to starvation is beneficial in the short term, as individuals tend to store energy rather than consume it, and the adverse effect persists when malnutrition improves, eventually causing metabolism dysfunction in adulthood [24, 25, 51].

Most studies have focused on the impact of early famine on MetS; however, there are scarce studies on the effects of famine exposure later in life. In this study, the strongest associations were observed between adolescence or adulthood famine exposure and the highest prevalence of MetS (OR 2.45, 95% CI 2.02, 2.97). Wang Z found that as the age of famine exposure increased, the OR of dyslipidemia was 1.80 (95% CI 1.26, 2.57), 1.75 (95% CI 1.17, 2.62), and 1.63 (95% CI 1.10, 2.42) in fetal, infant, and preschool exposure respectively, and the negative linear association was different from that observed in our study[5]. Wang Y reported that fetal exposure to famine was associated with a higher prevalence of diabetes after adjustment for sex (RR 2.11, 95% CI 1.01, 4.44) than famine exposure during childhood [4]. Chen C found a 17% increase in the visceral adiposity index (VAI) with fetal exposure to famine and a 13% increase in childhood exposure to famine compared to the unexposed group [28]. Yu C found that, among all childhood stages, the highest OR of MetS was 1.47 (95% CI 1.18, 1.84) during late childhood exposure [56]. Most studies suggest that fetal or infant undernutrition caused by famine could exert a more profound impact on metabolic functions. To date, there is no evidence supporting the highest rates of exposure in adolescence or adulthood. The mechanism underlying the positive association between a higher prevalence of MetS and famine exposure later in life remains unclear. We postulate that the variations may be caused by heterogeneity of the samples.

Several cross-sectional studies reported that earlier menarche age was inversely associated with MetS [18, 19, 57–59]. Furthermore, a meta-analysis showed that early age at menarche was associated with a higher risk of MetS with a pooled relative risk of 1.62 (95% CI 1.40, 1.88)[60]. Although the threshold for age at menarche varied, the overall trend was that MetS negatively correlated with age at menarche. Obesity may act as a mediator in the relationship between age at menarche and MetS prevalence [16, 59]. Obese children tend to develop an early onset of puberty and have increased insulin production, which promotes the release of gonadotropins and decreases the level of sex hormone-binding globulin, contributing to insulin resistance [18, 36, 61–65]. Evidence from animal models has shown that hyperinsulinemia leads to diet-induced obesity [47]. Hence, obese and early pubertal women tend to develop insulin resistance or hyperinsulinemia [16, 20, 60, 65]. Lee HS found that the risk of MetS increased significantly only at an age of menarche < 12 years and higher levels of triglycerides; the genome-wide association studies (GWAS) pathway, which contained amino-terminal kinase level pathways and stress-activated protein kinase signals, were also found to be associated with increased triglyceride levels among women with earlier menarche [66].

However, other studies have revealed that early or late menarche is correlated with a higher risk of MetS. In a prospective U.S. study involving 272 school girls followed up for 26 years, Glueck CJ observed a U-shape pattern of relationship between adult metabolic rate and menarche occurrence and found an adjusted odds ratio of early-late menarche of 3.43 compared to the standard menarche age group [20]. In our study, menarche at ≤ 15 years of age did not affect the risk of MetS, while menarche at ≥ 16 years of age increased the risk of MetS (RR 1.23, 95% CI 1.09, 1.39), which is consistent with the findings reported by Glueck CJ. In addition, several studies have demonstrated that early or late menarche is not correlated with MetS. In a study involving 3023 Korean women, Hwang YS demonstrated that earlier or later menarche was not related to increased MetS risk [67]. These findings were confirmed by Cho and Cui [22, 23]. Contrary to our results, a cross-sectional study of 1432 women in Bangladesh suggested that earlier age at menarche was associated with a lower level of fasting blood glucose compared to an older age at menarche [21].

Most studies have focused on the influence of MetS on separate factors including early menarche, age, and famine exposure. No studies have explored the combined effects of famine exposure and later menarche on the prevalence of MetS among females. Additionally, the combined effect of adolescent or adulthood exposure and menarche age ≥ 16 years was associated with the highest risk of MetS among all groups (OR 3.27, 95% CI 2.44, 4.38). Some potential mechanisms may explain the combined effect of famine exposure and late menarche age on the prevalence of MetS, which was stronger than the separate effects. Menstruation is an important indicator of the reproductive maturity of women. The gonadotropin-releasing hormone is synthesized by the hypothalamus and released into the portal circulation reaching the adenohypophysis which triggers the secretion of follicle-stimulating hormone and luteinizing hormone, which stimulates the release of ovarian estrogen and progesterone, leading to endometrial hyperplasia [60, 64–66, 68, 69]. Firstly, we assumed that famine exposure during adolescence or adulthood caused malnutrition. The reason for delayed age at menarche was usually associated with sickness, poor parenting, or malnourishment, these factors were accentuated as superimposed stress can lead to a reduction in estrogen as a protective hormone. Oestrogen promotes the liver synthesis of high-density lipoproteins, inhibits the synthesis of low-density lipoproteins, reduces cholesterol levels in the peripheral blood, and promotes bone matrix metabolism [36, 70–72]. Moreover, the protective effect occurred later than that in the group with an average age at menarche. Abnormal lipid metabolism is an essential factor in MetS, and some studies have shown that famine exposure can damage lipid profiles and increase the prevalence of dyslipidemia and MetS later in adulthood [20, 73, 74]. Secondly, late menarche means a shorter reproductive lifespan and premature menopause, particularly after experiencing famine during the three disasters in China. This study did not determine whether famine exposure belonged to the category of poor parenting. However, Demakakos P observed that the quality of the home environment was positively correlated with age at menarche in homozygous participants of the estrogen receptor α gene (ESR1) alleles, confirming the hypothesis of gene and environment interaction [75]. Thirdly, famine exposure during adolescence or adulthood is a powerful stressor that acts on the body, leading to the excessive breakdown of fat and proteins that can produce reactive oxygen species (ROS) in organelles such as the mitochondria [76, 77]. This oxidative stress further intensifies the excessive production of mitochondrial and endoplasmic reticulum (ER) ROS, leading to severe mitochondrial damage and ER stress, and becomes a catalyst for insulin resistance and hyperglycemia [24, 25, 78]. Furthermore, long-term famine, as a stressor, can change the bioequivalence and induce metabolic state instability, which is another reason for MetS [14]. Fourthly, accumulating evidence suggests that famine exposure can lead to several vitamin imbalances, and nutraceutical supplementation to counteract these imbalances in later life may play a significant role in women’s health. Previous studies found that famine exposure caused a decrease in vitamins C and D. Vitamin C is essential to improve erythrocyte osmotic fragility, and decreased oxygen content is an important etiologic factor in diabetic microvascular disease, which is a component of MetS. Vitamin D is discharged into the bloodstream after fasting and regulates metabolism in the body and deficiency has been associated with MetS [79, 80]. These cumulative effects may lead to severe malnutrition, impaired immunity, and microinflammation during adolescence. The release of pro-inflammatory factors can become a risk factor for diabetes and cardiovascular disease [81, 82].

## Conclusions

This cross-sectional study aimed to determine the separate and combined effects of famine exposure and age at menarche on the prevalence of MetS among elderly women. We conclude that late menarche and famine exposure are strongly associated with the prevalence of MetS. Among all the subgroups, individuals exposed to famine in adolescence or adulthood with an age of menarche ≥ 16 years had the highest prevalence of MetS. These findings support further expansion of MetS risk factors. Targeting elderly female populations with a history of exposure to famine at different stages of life can help prevent the development of MetS in later life.

### Strengths and limitations of the study

This study has several strengths. First, this study evaluated the separate and combined associations between famine exposure and age at menarche in MetS for the first time. Second, the effects of famine exposure on MetS at different stages of development were assessed, including fetal, childhood, and adolescence/adulthood exposure. Third, the effects of different ages at menarche (≤ 15 and ≥ 16 years) on MetS were assessed. A fully adjusted model was used to assess these effects, thereby avoiding potential confounding factors. Nonetheless, some limitations of this study should be noted. First, the effects of famine exposure in adulthood on MetS have not been thoroughly discussed and there is a paucity of evidence on this issue in the medical literature. Second, the samples of social backgrounds and demographic differences persisted. Third, famine survivors may have been healthier because of exposure to famine, and not every female participant experienced malnutrition. Fourth, there was an uncontrolled competing risk owing to the retrospective study design.

## Data Availability

The original data of the study are openly published as microdata at https://opendata.pku.edu.cn/dataverse/ CHARLS. The dataset generated by this study’s findings is available from the corresponding author on reasonable request.

## Abbreviations

Mets: Metabolic Syndrome
HDL: High-Density Lipoprotein
CHARLS: China Health and Retirement Longitudinal Study
ORs: Adjusted Odds Ratio
CI: Confidence Interval
OR: Odds Ratio
WC: Waist Circumference
SBP: Systolic Blood Pressure
DBP: Diastolic Blood Pressure
VAI: Visceral Adiposity Index
GWAS: Genome-Wide Association Studies
TC: Total Cholesterol
LDL: Low-Density Lipoprotein
ESR1: Estrogen Receptor α Gene
ROS: Reactive Oxygen Species
ER: Endoplasmic Reticulum
GnRH: Gonadotropin-Releasing Hormone
DNA: Deoxyribonucleic Acid
FSH: Follicle-Stimulating Hormone
LH: Luteinizing Hormone
IGF2: Insulin-Like Growth Factor 2
